# Literary Notes

**Published:** 1896-03

**Authors:** 


### Literary Notes.

THE report of the Commissioner of Education, Vol. II., soon to issue, will contain a chapter on Medical Education by A. Erskine Miller, that cannot fail to interest all physicians who believe in improved methods of teaching. Prof. Miller says that New York has gone further than any other state to prevent the licensing of persons not qualified. Let New York continue to maintain this proud position, by advancing the standard and not lower it as has been urged by some. Four years will soon be the minimum limit of collegiate study in this State.

---

DR. H. BRONSON GEE has resigned the editorship of the *New York State Medical Reporter*, and the management has secured the services of Dr. Charles Wilson Ingraham, of Binghamton, to take charge of the editorial department of this publication.

---

SEVERAL new journals have appeared since the beginning of the new year. Among them we may mention *Langsdale's Lancet*, Kansas City, Mo., John M. Langsdale, editor and owner; *The Clinical Recorder*, New York City, Wm. S. Gottheil, M. D., editor, and *The Medical Council*, Philadelphia, J. J. Taylor, M. D., editor and publisher. We wish them all success.

---

JUST as we are printing this page the *State Hospitals Bulletin* presents itself. It is to be a quarterly report of clinical and pathological work in the state hospitals for the insane, and is published at Utica by authority of the state commission in lunacy. The president, the hospital superintendents and director of the pathological institute are the editors. Drs. Wise, Pilgrim and Talcott compose the editorial committee and the assistant physicians and medical internes of the several state hospitals are collaborators. The first number is a handsome specimen of medical magazine making.

---

*L'Union Medicale du Canada* appeared in a new dress at the beginning of the year. It is enlarged and in every way much improved. Our excellent contemporary begins its twenty-fifth year in good form.

---

THE *Sanitary Era* has put itself into a new dress, changed its name and form, and will hereafter be known as *Modern Medical Science*. This is a good title and we hope it will indicate the contents of the magazine.

---

P. BLAKISTON SON & COMPANY, of Philadelphia, announce a book on Appendicitis, by John B. Deaver, M. D., assistant professor of applied anatomy, University of Pennsylvania; assistant surgeon to the German Hospital, etc. The book will be arranged in a practical and systematic manner. The history, etiology, symptoms, diagnosis, operative treatment, prognosis and complications of this disease will be given in the order named. It will contain about forty illustrations of methods of procedure in operating and typical pathological conditions of the appendix, the latter being printed in colors.

---

DON'TS for consumptives, or the scientific management of pulmonary tuberculosis, is the title of a book which, under the authorship of Dr. Charles Wilson Ingraham, is about to be issued by the Medical Reporter Publishing Company, of Rochester, N. Y. Price, $1.75.

